# Electrophysiological signatures predict the therapeutic window of deep brain stimulation electrode contacts

**DOI:** 10.1038/s41746-025-02089-w

**Published:** 2025-10-29

**Authors:** Fayed Rassoulou, Abhinav Sharma, Alexandra Steina, Markus Butz, Christian J. Hartmann, Bahne H. Bahners, Jan Vesper, Alfons Schnitzler, Jan Hirschmann

**Affiliations:** 1 Institute of Clinical Neuroscience and Medical Psychology, Medical Faculty and University Hospital Düsseldorf, Heinrich Heine University Düsseldorf, Düsseldorf, Germany; 2 MRC Brain Networks Dynamics Unit, University of Oxford, Oxford, UK; 3 Nuffield Department of Clinical Neurosciences, University of Oxford, Oxford, UK; 4 Center for Movement Disorders and Neuromodulation, Department of Neurology, Medical Faculty and University Hospital Düsseldorf, Heinrich Heine University Düsseldorf, Düsseldorf, Germany; 5 Department of Functional Neurosurgery and Stereotaxy, Medical Faculty and University Hospital Düsseldorf, Heinrich Heine University Düsseldorf, Düsseldorf, Germany

**Keywords:** Parkinson's disease, Predictive markers

## Abstract

Deep brain stimulation (DBS) is an effective treatment for Parkinson’s disease. Identifying the optimal parameters is a complex task. Here, we investigated whether electrophysiology, combined with machine learning, can support contact selection. We applied tree learning to resting-state magnetoencephalographic and local field potential recordings from the subthalamic nucleus (STN). STN power and STN-cortex coherence in various frequency bands served to predict the therapeutic window. The model successfully predicted therapeutic windows in the original (*r* = 0.45, *p* < 0.001, *N* = 45) and in an independent cohort (*r* = 0.30, *p* < 0.001, *N* = 8). It relied mostly on fast (>35 Hz) subthalamic activity and on STN-cortex coherence in several bands. Furthermore, it was able to order contacts such that the optimal contact can be found faster. Our study demonstrates the feasibility of predicting therapeutic windows from electrophysiological features and could contribute to automated contact selection in the future.

## Introduction

Deep brain stimulation (DBS) is a well-established therapy for neurological movement disorders such as Parkinson’s disease (PD)^1^. PD is characterized by degeneration of dopaminergic neurons in the midbrain, leading to motor impairment and to pathological oscillatory patterns in the basal ganglia^2^. Among these structures, the subthalamic nucleus (STN), a key regulator of motor function^3^, has become an established target for DBS^4^.

Although stimulation of the STN is effective, achieving optimal symptom relief can be challenging in some patients, due to the anatomical complexity of the midbrain. Choosing a suboptimal contact for stimulation, for example, might lead to the activation of non-target brain areas, causing side effects. These, in turn, forbid the usage of amplitudes high enough to fully suppress motor symptoms, i.e, they limit the therapeutic window. Accordingly, one major aim in DBS programming is to find the electrode contact with the largest therapeutic window, to be used for chronic stimulation. This is achieved through the so-called monopolar review, a clinical procedure involving incremental increases of stimulation amplitude while assessing symptom relief as well as side effects^5^.

Monopolar reviews are both time-consuming and tedious because each patient requires a tailored approach^6–8^. The complexity of this task has increased further with the development of advanced DBS systems that allow for thousands of possible parameter combinations^9,10^. As decision-support systems are increasingly being adopted across clinical domains^11^, it is logical to explore how machine learning techniques could help speed up the identification of optimal DBS settings. These techniques have already started to show promise in the DBS field (see refs. ^12,13^ for a review).

The majority of machine learning studies addressing DBS programming come from the imaging domain^14–16^. A landmark study by Roediger et al., for example, demonstrated the validity of automated programming in a clinical trial^7^. The authors developed and evaluated the so-called StimFit algorithm, which models the relationship between electrode location and DBS settings on the one hand and clinical outcome on the other hand through linear mixed effect models, and selects optimal DBS settings by means of gradient descent optimization^10^. Applying this strategy to PD patients in a randomized, double-blinded trial, the authors demonstrated non-inferiority of automated programming compared to conventional programming. Other studies applied machine learning to kinematic measurements to improve DBS programming^17–20^. Sarikhani and colleagues, for example, have recently optimized DBS programming for tremor suppression based on smartwatch-accelerometery^19^. Automated parameter selection by means of Gaussian process regression achieved similar tremor suppression as expert clinicians.

Besides imaging and kinematics, electrophysiology has been a major focus of this research. Neural oscillations, as obtained via electroencephalography, magnetoencephalography (MEG) or local field potential (LFPs) recordings, are of particular interest to the field because they encompass relevant neural signatures associated with Parkinsonian symptoms. STN beta power, for example, is well known to correlate with bradykinesia and rigidity^21–24^. Due to this robust relationship, it is commonly used as a feedback signal for automated DBS parameter adjustments in closed-loop stimulation^25–27^. Moreover, it has recently been validated for contact selection^28,29^.

However, STN beta power is not the only signal related to PD symptoms. In fact, the literature bears a wealth of candidate markers, many of which are symptom or state-specific. This includes finely-tuned and broadband gamma oscillations^30–33^, oscillations in the tremor range (4–8 Hz)^34–36^ and high-frequency oscillations^37–39^, just to name a few. Given this rich set of potentially informative signals, it seems suboptimal to base decisions on a single marker. A more holistic approach would be to leverage multivariate tools that base decisions on a large set of variables.

Here, we use such a strategy to predict therapeutic windows from various electrophysiological markers. Based on the STN’s fundamental contribution to PD pathophysiology, we take an STN-centric view and consider STN power in various frequency bands. Subthalamic activity alone, however, might not be sufficient for optimizing DBS parameters. Recent research suggests that cortical signals are a major asset when inferring clinical parameters of interest^40^. Thus, we further include STN-cortex coherence, a functional connectivity metric capturing subthalamo-cortical interactions.

## Results

We analysed resting-state data from two prior studies^41–43^, which involved 26 and 19 PD patients, respectively (Table 1). All patients were implanted with externalized DBS electrodes, allowing for simultaneous MEG-LFP measurements. Recordings took place the day following DBS electrode implantation and after an overnight withdrawal from dopaminergic medication. The data from this combined cohort were used to train an extreme gradient boosting model^44^ predicting the clinical utility of each electrode contact based on the neural oscillations recorded at this contact, and their synchrony with cortical oscillations. Clinical utility was quantified by the normalized therapeutic window, i.e., the difference between clinical effect and side effect threshold, as obtained from monopolar review (see Methods). Model performance was assessed using a leave-one-electrode-out (LOEO) cross-validation scheme. Subsequently, we validated the model in an independent cohort (Table 2).

**Table 1:** Patient information for the cohort used for leave-one-out cross-validation

Id	Study	Age [y]	Sex	Electrode type	Monopolar review [day after surgery]	Disease duration
S01	1	75	f	MDT 3389	36	21
S02	1	70	m	MDT 3389	6	11
S03	1	64	f	MDT 3389	8	14
S04	1	62	f	MDT 3389	6	15
S05	1	54	f	MDT 3389	16	10
S06	1	47	m	MDT 3389	5	10
S07	1	68	m	MDT 3389	6	11
S08	1	66	m	MDT 3389	6	8
S09	1	69	m	MDT 3389	6	6
S10	1	70	m	MDT 3389	12	11
S11	1	60	m	MDT 3389	6	6
S12	1	69	m	MDT 3389	6	1
S13	1	67	m	MDT 3389	6	6
S14	1	53	m	MDT 3389	6	11
S15	1	61	f	MDT 3389	9	10
S16	1	52	m	BSC Vercise Standard	7	9
S17	1	53	m	BSC Vercise Standard	8	6
S18	1	44	m	MDT 3389	6	5
S19	1	50	m	MDT 3389	10	18
S20	1	44	f	MDT 3389	372	9
S21	1	65	m	MDT 3389	6	4
S22	1	69	m	MDT 3389	7	12
S23	1	61	f	MDT 3389	6	3
S24	1	72	m	MDT 3389	6	14
S25	1	66	m	MDT 3389	6	12
S26	1	75	f	MDT 3389	7	14
S27	2	69	m	ABI 6662	113	12
S28	2	65	m	ABI 6662	6	6
S29	2	56	m	ABI 6662	498	13
S30	2	62	f	ABI 6662	6	13
S31	2	70	m	ABI 6662	671	11
S32	2	45	m	ABI 6662	125	13
S33	2	60	f	ABI 6662	419	6
S34	2	59	f	ABI 6662	100	9
S35	2	66	f	ABI 6662	6	10
S36	2	72	f	ABI 6662	6	3
S37	2	69	m	ABI 6662	103	10
S38	2	54	m	ABI 6662	5	3
S39	2	55	f	ABI 6662	50	4
S40	2	41	m	ABI 6662	6	7
S41	2	46	m	ABI 6662	357	6
S42	2	59	m	ABI 6662	225	15
S43	2	58	m	ABI 6662	49	8
S44	2	62	f	ABI 6662	6	2
S45	2	59	m	ABI 6662	58	10
mean	—	61	—	—	75	9
median	—	62	—	—	7	10
sd	—	9	—	—	149	4

Age refers to the date of measurement.

*MDT* Medtronic, *BSC* Boston Scientific, *ABI* Abbott Infinity, *m* male, *f* female.

**Table 2:** Patient information for the independent cohort

Id	Study	Age [y]	Sex	Electrode type	Monopolar review [day after surgery]	Disease duration
S01	3	68	m	ABI 6662	6	13
S02	3	57	m	MDT SenSight	6	6
S03	3	66	m	BSC Vercise Cartesia	6	4
S04	3	68	m	ABI 6662	51	9
S05	3	58	m	ABI 6662	7	7
S06	3	64	f	BSC Vercise Cartesia	51	5
S07	3	81	m	ABI 6662	82	7
S08	3	49	m	ABI 6662	57	11
mean	—	64	—	—	33	8
median	—	65	—	—	29	7
sd	—	9	—	—	28	3

Age refers to the date of measurement.

*MDT* Medtronic, *BSC* Boston Scientific, *ABI* Abbott Infinity, *m* male, *f* female.

### Model performance

**Fig. 1:**
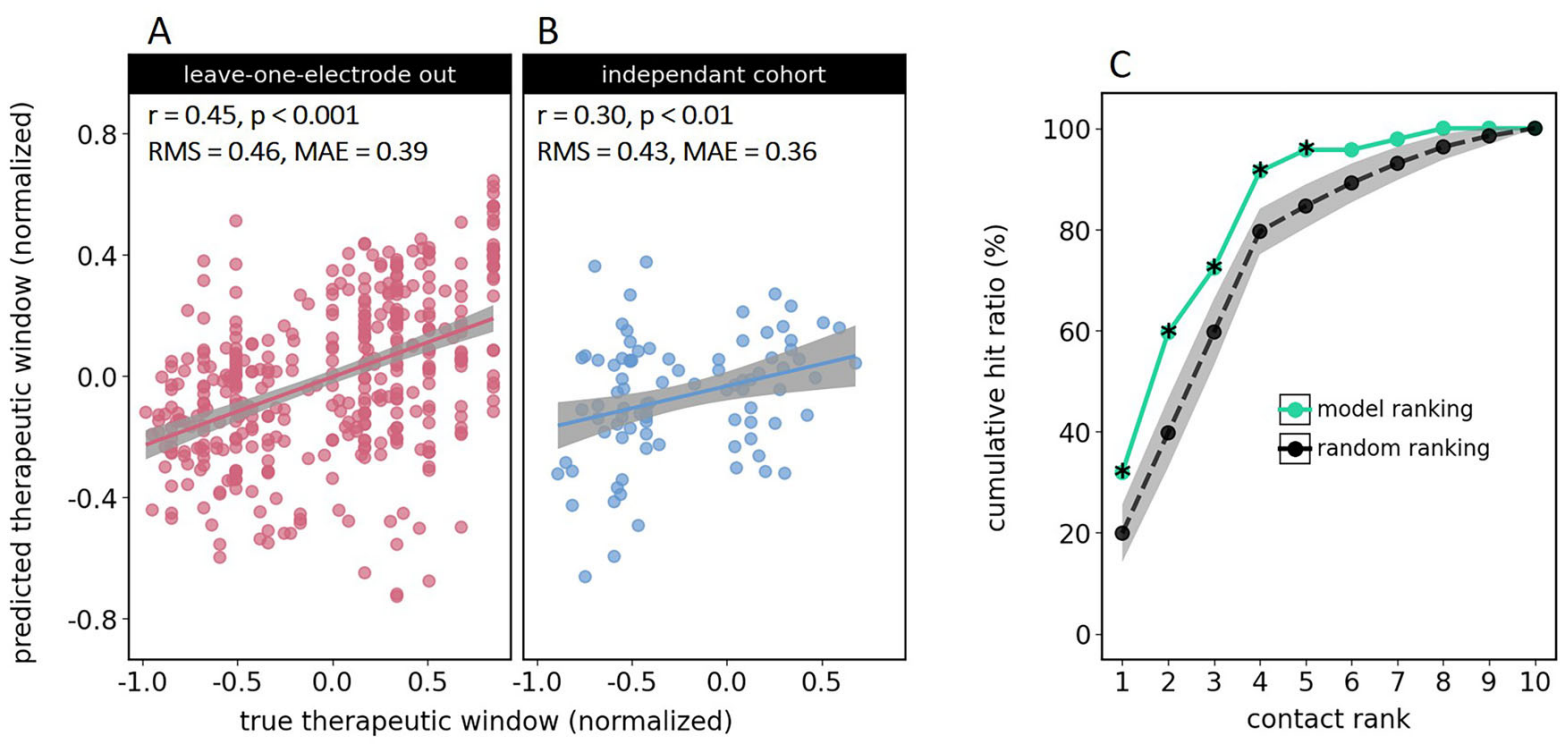
Model performance. **A** Scatter plots displaying the relationship between actual and predicted therapeutic windows for the leave-one-electrode-out approach. Gray shaded areas represent 95% confidence intervals. **B** Same as (**A**), but showing predictions generated for the independent cohort. **C** Cumulative hit ratio for the model’s ranking of DBS electrode contacts (green) and the average hit ratio resulting from random ranking (black). The gray shaded area represents the mean ± 1 standard deviation. Asterisks (*) indicate above-chance performance (*p* < 0.05).

**Fig. 2:**
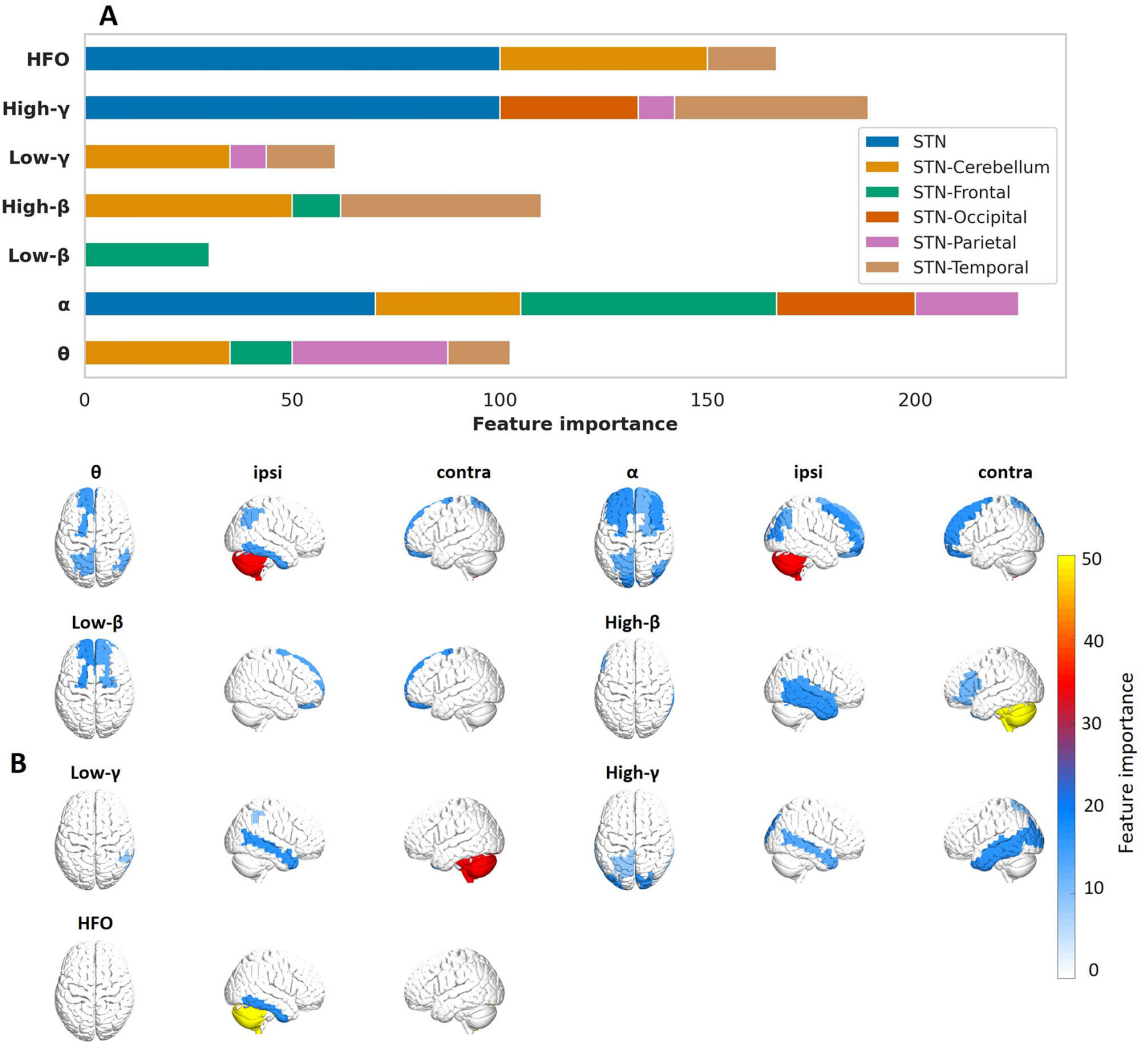
Distribution of feature importance across brain regions and frequency bands. **A** The bar plot illustrates the importance of each feature, grouped by brain region (indicated by colors) and frequency band (θ, α, β, γ, HFO). The length of each bar represents the relative contribution of each feature. Feature importance was quantified by selection frequency across leave-one-out cycles, normalized by the number of brain areas per lobe. **B** 3D topography of STN-cortex coherence features (parcellated).

The model’s ability to predict the therapeutic window was quantified by the correlation between actual and predicted values. As shown in Fig. 1A, the LOEO validation yielded a positive correlation (*r* = 0.45, *p* < 0.001), indicating that the model successfully captured the relationship between neural oscillations and therapeutic windows. To account for repeated measures per electrode, we also fitted a linear mixed effect model as an alternative performance measure, yielding similar results (β = 0.312, CI = [0.093, 0.532], *p* < 0.01; Supplementary Table S3). When we applied a model trained on the entire original cohort to a new set of patients, the positive correlation remained (*r* = 0.30, *p* < 0.01), demonstrating the model’s ability to generalize to unseen data (Fig. 1B). This model used the features most frequently selected in the LOEO procedure (Fig. 2A).

Figure 1C demonstrates the model’s effectiveness in ranking electrode contacts according to their therapeutic window. It compares two scenarios in which the electrode contacts are first ordered, either randomly or according to the predicted therapeutic window (model-based), followed by a sequential search for the “optimal” contact, i.e., the contact chosen by the clinician. The cumulative hit ratio quantifies, for each rank *i*, the fraction of electrodes for which the optimal contact got ranked *i*th or better. The steepness of the curve thus indicates how fast one would find the optimal contact using a given ranking. The model-based ranking outperformed the vast majority of random rankings (*p* < 0.05), confirming the model’s ability to inform the search for the optimal contact. These results indicate practical utility in guiding DBS programming decisions.

### Feature importance

#### Full model

Feature importance analysis was conducted to identify the frequency bands and brain regions most relevant for predicting the therapeutic window. Using the Boruta algorithm during each iteration of the LOEO cycle (see Methods), we aimed to retain only the features that contributed significantly to model predictions. Thus, the number of times a feature got selected is indicative of its importance. Here, we used this quantity to assess feature importance for the full model, considering both STN power and STN-cortex coherence features.

From the initial set of 217 features (31 brain regions * 7 frequency bands), a total of 36 were selected consistently across iterations. Figure 2 illustrates the selection frequency for different features, grouped by frequency band (θ, α, β, γ, and HFO) and brain region. STN power features were particularly influential, particularly in higher frequency bands (γ and HFO). When applied to the subset of patients exhibiting one or more prominent beta peaks, STN beta power emerged as an important feature, indicating that subthalamic beta power is helpful when present (Supplementary Fig. S5).

Regarding STN-cortex coherence, the features spanned a broad spectrum, indicating that STN connectivity to multiple regions, in various frequency bands, contributed to the prediction of therapeutic windows. Notably, STN connectivity with the cerebellum, the temporal lobe, the superior frontal and superior parietal cortex were among the most important predictors.

#### Reduced feature sets

An alternative way of addressing feature importance is to exclude a subset of features and to reassess prediction performance. Here, we used this strategy to investigate the contribution of STN power vs. STN-cortex coherence, the importance of the cerebellum, and the possibility of obtaining accurate predictions when using only subthalamic and primary sensorimotor features.

A model trained on STN power alone yielded a lower performance than the full model (*r* = 0.22, *p* < 0.001; Supplementary Fig. S1A). In contrast, a model trained on STN-cortex coherence achieved a correlation comparable to that of the full model (*r* = 0.40, *p* < 0.001; Supplementary Fig. S1C). Despite the relatively strong performance, the cumulative hit ratio analysis did not indicate any ability to speed up the search for the optimal contact (Supplementary Fig. S1D), emphasizing the advantage of combining STN power with STN-cortex coherence.

Excluding the cerebellum from the feature set (Supplementary Figs. S2A, B and S3), or all cortical areas except sensorimotor cortex (Supplementary Fig. S2C, D), worsened predictions, but still yielded significant correlations between predicted and actual therapeutic windows (*r* ≥ 0.32, *p* < 0.001).

#### Enhanced feature set

In order to test whether the addition of anatomical information would improve predictions we tested an enhanced feature set that included the best-performing electrophysiological features (Fig. 2A) and the distance of the contacts’ center to a published anatomical sweet spot for STN DBS (x = 12.58, y = −13.41, z = −5.87^45^). We tested both feature sets in the LOEO framework and found that the anatomical information improved both the prediction performance and the cumulative hit ratio (Supplementary Fig. S4), highlighting the merits of combining electrophysiology and anatomy.

## Discussion

In this proof-of-concept study, we show that STN power and STN-cortex coherence allow for predicting a contact’s therapeutic window, demonstrating the potential of electrophysiological markers for contact selection. No single feature dominated the prediction; rather, a combination of features across different brain regions and frequency bands—particularly in the alpha, high-gamma, and HFO range— facilitated the prediction of therapeutic windows. Predictions could be improved by adding anatomical information. Overall, these results highlight the importance of integrating information from multiple sources rather than relying on isolated markers.

Using electrophysiological features, we could successfully predict therapeutic windows in both the original and the independent cohort. The generalization to a new set of patients is particularly important for any future application to be developed based on this study, because the overarching goal is to gain a time advantage, requiring that predictions are based on previously recorded data. This sort of generalization is difficult to achieve in clinical neuroscience, due to the high between-subject and between-study variability of electrophysiological recordings^46^ and due to the numerous methodological caveats, that can lead to overfitting^47^. Our model likely profited from the fact that the original cohort included two distinct datasets^41–43^, forcing the model to focus on robust feature-outcome relationships. In addition, the features themselves appear to be rather robust. The basic, frequency-dependent layout of STN-cortex coherence has been replicated in numerous studies, indicating stability^41,48–51^. Accordingly, we observed a high similarity between the original cohort and the independent cohort in this study (Fig. 4).

The main value of machine learning in medicine is to provide clinically useful tools. For the current study, the utility lies in the time advantage that our approach might bring to the tedious, trial-and-error search for the best DBS contact. Our study sets the ground for such a tool by demonstrating that predicting therapeutic windows from electrophysiological signatures is possible in principle. Moreover, we provide evidence of an advantage over random search, which is the current standard, in our cumulative hit ratio analysis. Whether or not the approach really saves time in practice, however, needs to be established in a prospective study.

Besides producing useful tools, machine learning in medicine has the potential to uncover previously unknown relationships between measured signals and clinical outcomes. A machine learning model monitors a vast number of variables simultaneously and, unlike humans, is not biased by expectations^52^. Hence, investigating the features important to a successful machine learning model can provide scientific insights. In this context, it is important to keep in mind that the term “importance” refers to the model’s internal computations, not to the process of interest. Accordingly, a model might rely on features that are unrelated to the underlying causal mechanisms^53^. That being said, we continue with discussing the most important features identified in this study.

In general, STN power was important to the model, particularly in the alpha, high-gamma and HFO range. This result tallies with a study by Shah et al., who used Lasso-regression to predict therapeutic windows from LFPs recorded intraoperatively in the resting-state. Matching the current results, the authors identified STN high-gamma and HFO power as the most predictive features^54^. The importance of fast STN oscillations is further corroborated by recent studies on adaptive DBS at home, which found that stimulation-entrained gamma oscillations best reflect motor symptom fluctuations^32,55^. Our previous study, which aimed at predicting the motor symptom improvement achieved with the optimal contact, lends further support to the current findings: in the STN power model, STN alpha power ranked second, and HFO power ranked third^56^. The best STN feature in that study, however, was high-beta power, in line with previous reports^57,58^, prompting the question of why STN beta power did not emerge as a dominant feature here.

The reasons could be methodological, as both the target variable (therapeutic window vs. UPDRS sum score improvement) and the method for feature selection (BORUTA vs. Shapley values) differed across studies. Further, subthalamic beta and gamma power are anti-correlated^59^, and feature selection in a set of correlated features is arbitrary to some degree. Beyond that, there might also be neurobiological reasons. STN beta power is primarily related to akinesia and rigidity^23^. For the monopolar review, however, tremor is usually the preferred target symptom, if present, because it is an easy readout. Thus, the review data might be less related to beta power than clinical scores with a stronger emphasis on akinesia and rigidity, such as the UPDRS. Further, in this particular dataset, there were many contacts lacking a clear beta power peak, presumably due to the stun effect^46^. This raises the question whether beta power is helpful when present. Indeed, we found STN beta power to be important in the set of patients exhibiting a clear beta peak.

In line with our previous work^56^, we find that STN-cortex connectivity provides valuable information for predicting DBS effects. The coherence-only model performed better than the STN-only model and both performed worse than the combined model, indicating that STN power and STN-cortex coherence are complementary sources of information. With respect to STN-cortex coherence, our model mostly relied on low-frequency coupling (theta to low-beta) to bilaterally symmetric, medial, fronto-parietal regions. Further, it relied on coherence with the ipsilateral temporal cortex, and on coherence with the contralateral cerebellum (both not frequency-specific). In the following, we briefly discuss each of these couplings.

Low-frequency coherence with medial frontal areas was particularly important to the model. This is interesting because the STN has dense structural connections with the medial frontal cortex^60^. Structural connectivity forms the basis of functional connectivity. Accordingly, recent studies demonstrate that structural connectivity and STN-cortex coherence correlate in a frequency- and area-specific fashion^61,62^. Structural connectivity is also a major predictor of the DBS effect^63^, with the set of important fibers varying systematically across diseases^60^ and symptoms^64^. Taken together, these considerations suggest that STN-cortex coherence might be useful for contact selection because it contains information about the structural connections within reach of a given contact. Some of these connections might be critical to the DBS effect, as they convey clinically relevant, cortical afferents^65,66^ or because they mediate remote cortical effects of DBS^67–69^. Finally, coherence and power might be indicative of fibers or regions that should be avoided, such as the pyramidal tract^70^. Despite being closely related, electrophysiology and anatomy are distinct sources of information. As demonstrated here, their combination can improve predictions—a finding that might inspire future applications.

The ipsilateral temporal lobe is known to be one of the hot spots of STN-cortex coherence, primarily in the alpha band^41,48,49^. This coupling is not STN-specific, but has been reported for numerous DBS targets, such as the ventral thalamus^71^, the internal pallidum^72^ and the nucleus basalis of Meynert^73^. Neither the anatomical underpinnings nor the functional significance are well understood to date. Yet, its occurrence in a number of different diseases suggests a physiological rather than a pathological nature.

The strong contribution of the cerebellum is likely based on structural connections between the STN and cerebellum, which are reciprocally connected via a disynaptic pathway involving the pontine nuclei^74^. As expected, due to fiber decussation, we observed the strongest contribution from the cerebellar hemisphere contralateral to the STN. Both, the basal ganglia and cerebellum, are important hubs for motor learning^75^ and tremor^76^. Modulating their functional interactions might thus be a critical aspect of DBS, and contacts allowing for this modulation might be better suited than others. In line with this idea, we observed that removing cerebellar features from the input impaired model performance.

Interestingly, there appears to be some segregation between the areas with the strongest coherence (Fig. 4) and the areas with the most informative coherence (Fig. 2), most strikingly in the beta band. The ipsilateral sensorimotor cortex is strongly coupled to the STN in the beta band, but that coupling does not seem to be very useful for predicting the therapeutic window (current study), nor for predicting the UPDRS improvement^56^. This finding aligns with a study on DBS effects on STN-cortex coherence. Standard 130 Hz DBS markedly reduced beta-band coherence between sensorimotor cortex and STN, but this reduction did not correlate with the concurrent reduction of symptoms^50^, suggesting that these two consequences of DBS are largely independent.

With respect to the time delay between electrophysiological measurements and clinical assessment, our study demonstrates that recordings obtained 1 day after surgery can predict the contact selected during monopolar reviews conducted between 5 and 671 days post-surgery, suggesting relative stability of neural oscillations over time. This perspective is supported by work from Garcia et al., who reported that subthalamic oscillations recorded intraoperatively can predict clinically effective contacts up to 27 months later^28^. Although these and similar findings^77^ indicate that early recordings can be useful for guiding future programming decisions, it is to be expected that prediction performance improves with decreasing the delay between electrophysiological recording and monopolar review, as more immediate measurements better capture the neural dynamics predominating at the time of clinical assessment. Here, most of the reviews were performed <30 days after surgery, ensuring temporal proximity between the electrophysiological recordings and the monopolar review. Using early reviews, however, comes at the disadvantage of the stun effect masking some of the clinical effects. This is the reason why we observed many negative therapeutic windows, indicating the presence of side effects in the absence of clinical effects (Fig. 1). Ideally, reducing the delay between recording and review should be achieved by shifting the recordings to a later stage. This is not possible when using externalized leads, but can be achieved with intracranial sensing^78^.

We acknowledge that MEG is not widely available, limiting the translation of our approach. Alternative setups, such as combining sensing-capable DBS stimulators with EEG or electrocorticography^55,62,79^ might be better suited for clinical application. This would require training a new model in a new cohort due to the differences with respect to channels and signal-to-noise ratio. Irrespective of the hardware, the procedure would involve obtaining a short electrophysiological measurement, feature computation and the prediction of therapeutic windows based on a pre-trained group model, rather than an individualized model. Contacts scoring very low might be omitted in the monopolar review, saving valuable time. Alternatively, contacts that were not initially expected to be useful, e.g., because they rarely yield good outcomes, might be explored with more rigor when scoring high. In this sense, our approach, the feasibility of which we demonstrated here, has the potential to speed up and improve the standard monopolar review.

In conclusion, this study demonstrates that STN power and STN-cortex coherence can be used to predict the width of the therapeutic window, the key criterion for contact selection. Our approach has the potential to reduce the duration of DBS programming. Moreover, our study confirms the utility of synchronized oscillations for generating clinically relevant predictions.

## Methods

### Patients

We analysed resting-state data from two prior studies conducted at the University Hospital Düsseldorf^41–43^, which involved 26 and 19 PD patients, respectively, with an average age of 60.73 years (SD = 9; Table 1). All patients were implanted with DBS electrodes, following standard clinical procedures, and took part with written informed consent according to the Declaration of Helsinki. Recordings took place the day following DBS electrode implantation and after overnight withdrawal from dopaminergic medication (Med OFF). The data from this combined cohort were used to train a machine learning model predicting the therapeutic window for each electrode contact based on the neural oscillations recorded at this contact, and their synchrony with cortical oscillations. The performance of this model was assessed using a leave-one-electrode-out (LOEO) cross-validation scheme. Subsequently, we validated our model in an independent cohort (Table 2) consisting of eight PD patients (M = 64; SD = 9), measured for the current study. Resting-state recording durations varied across cohorts (LOEO cohort 1: M = 263 s, SD = 56 s; LOEO cohort 2: M = 540 s, SD = 68 s; independent cohort: M = 385 s, SD = 106 s).

The experimental protocols were approved by the Ethics Committee of the Medical Faculty of Heinrich Heine University Düsseldorf for both the original cohort (no. 3209 and 5608) and the independent cohort (no. 2021-1587—Andere Forschung, Erstvotierend).

### Electrophysiology

Patients were recorded using a 306-channel MEG system (VectorView, MEGIN), while simultaneously measuring LFPs from the STN via externalized leads. The DBS electrodes were connected to the integrated EEG amplifier via non-ferromagnetic extension cables, minimizing magnetic interference. In addition to LFPs, we recorded vertical and horizontal electro-oculograms and electromyograms of the extensor and flexor muscles of both forearms. In our analyses, these were solely used for artifact detection. All signals were sampled by the same acquisition system, at a rate of 2 kHz [LOEO cohort 1 and independent cohort; **42**] and 2.4 kHz [LOEO cohort 2, **43**], respectively.

LFP signals were referenced to a surface electrode on the left mastoid and re-referenced post-hoc according to an average reference scheme. Within each electrode, we computed the mean across LFP channels and subtracted it from each signal.

#### Preprocessing

LFP and MEG data underwent visual artifact screening. Channels with a very low signal-to-noise ratio were marked as bad channels. On average, this led to the exclusion of 6.26 ± 6.67 (SD) MEG channels and 1.67 ± 1.81 electrode contacts per patient. Segments containing movement artifacts were likewise excluded from analysis. On average, this led to the exclusion of 13.66 s ± 24.80 s of data per patient. Data were segmented into 2 s windows with 50% overlap, resulting in a frequency resolution of 0.5 Hz.

#### Feature extraction

**Fig. 3:**
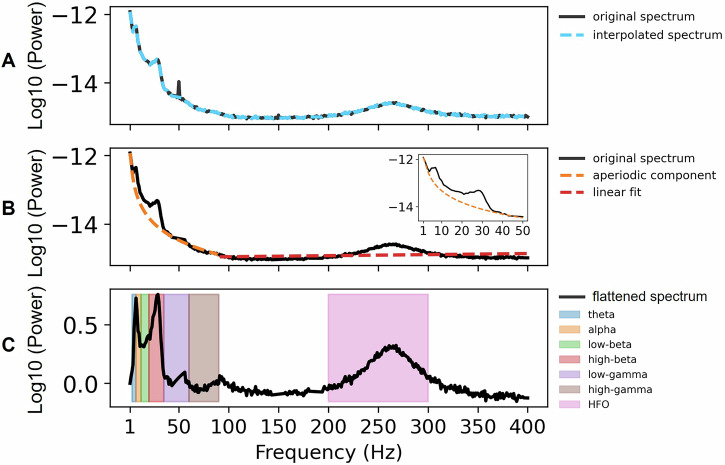
Subthalamic nucleus power feature extraction. **A** Power spectrum before (black) and after removing power line noise (blue). **B** Removal of the aperiodic component (1–90 Hz) and the linear trend (>90 Hz). **C** Preprocessed power spectrum. Frequency bands are color-coded.

Feature extraction details have been described in ref. ^56^. In brief, STN power features were computed for each integer frequency from 1 to 300 Hz using Welch’s method. To remove line noise (50 Hz) and its harmonics, power within ±7 Hz from the harmonics were replaced with surrogate values obtained through linear interpolation (Fig. 3A). The aperiodic (1/f) component of the LFP power spectra was removed using the fitting oscillations and one-over-f algorithm (FOOOF)^80^, applied up to 90 Hz. For frequencies above 90 Hz, we applied linear de-trending (Fig. 3B). The periodic component was retained, and power was averaged within eight frequency bands of interest: theta (3–7 Hz), alpha (8–12 Hz), low-beta (13–20 Hz), high-beta (21–35 Hz), low-gamma (36–60 Hz), high-gamma (60–90 Hz), and high-frequency oscillations (HFO; 200–300 Hz). The band definitions include the upper and the lower limit. LFP-MEG coherence was calculated for each frequency band using the multitaper method^81^ and source-localized through Dynamic Imaging of Coherent Sources^82^. The beamformer grid comprised 567 locations on the cortical surface, which were subsequently subdivided into 30 regions of interest based on the automatic anatomic labeling (AAL) atlas^83^ (Supplementary Table S2). We spatially normalized coherence by z-scoring across parcels. We used all available data for feature computation to maximize feature quality.

**Fig. 4:**
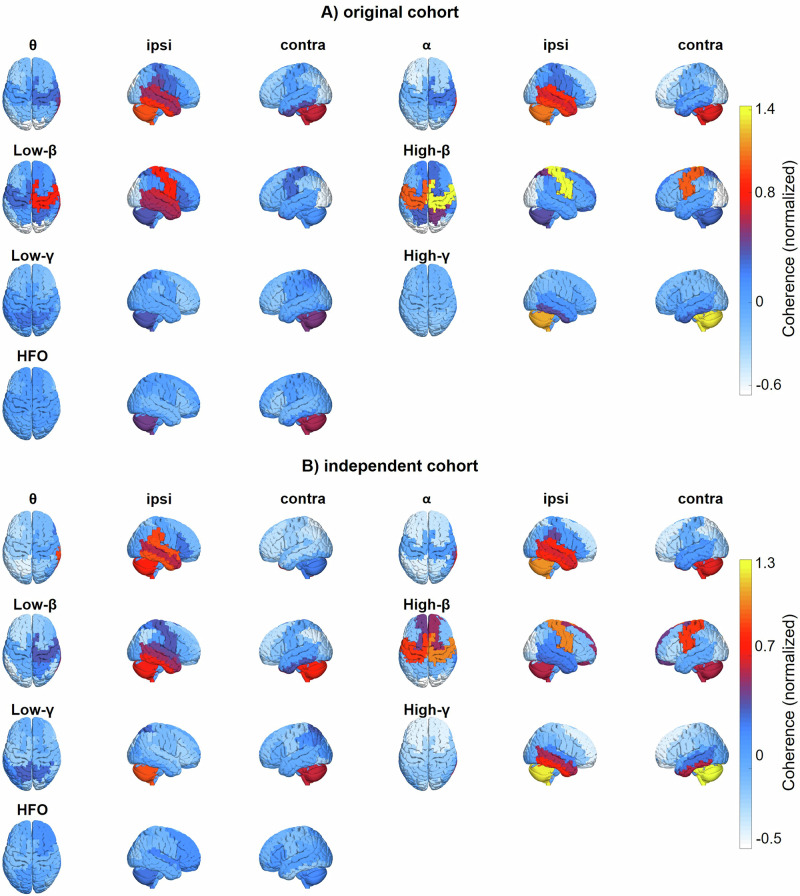
STN-cortex coherence. Source-reconstructed STN-cortex coherence for different frequency bands (θ, α, low-β, high-β, low-γ, high-γ, and HFO), projected onto a 3D template brain (parcellated). The topographies were normalized by means of spatial z-scoring before computing the group average. **A** Original cohort. **B** Independent cohort. The terms ipsilateral (ipsi) and contralateral (contra) refer to the DBS electrode.

When constructing group-average maps of coherence (Fig. 4), we mirrored coherence maps for electrodes implanted in the left STN across the mid-sagittal plane, aligning the hemisphere ipsilateral to the LFP recording site to the right hemisphere of the brain template.

#### Monopolar review

The aim of the monopolar review is to identify the optimal electrode contact for chronic stimulation. During the monopolar review, an experienced movement disorder neurologist tested each electrode contact in a monopolar configuration, i.e., with the contact as cathode and the neurostimulator case as anode. Stimulation amplitude was gradually increased in steps of 0.5 or 1 mA until reaching either the highest amplitude tolerated by the patient (side-effect threshold) or the highest amplitude considered safe (5 or 6 mA). At each step, the therapeutic and side effects of DBS were documented. The minimum amplitude required for a sustained clinical benefit was defined as the therapeutic effect threshold, and the electrode contact with the widest therapeutic window, defined as the difference between the side-effect threshold and therapeutic effect threshold, was chosen for chronic stimulation. Frequency and pulse width were kept constant at 130 Hz and 60 µs, respectively, and adapted later if required.

For each patient, the monopolar review was performed between 5 and 7 days after surgery for initial treatment and repeated between 4 and 13 weeks after surgery. Not all reviews encompassed the complete set of electrode contacts, including all contacts, all segments and ring modes. Accordingly, the number of contacts tested varied across patients (training cohort: median: 8, SD: 4.4, minimum 3, maximum 20; independent cohort: median: 13, SD: 6, minimum 8, maximum 20). In case several reviews were available per patient, we included the review with the highest number of contacts tested to maximize the amount of training data.

Some of the monopolar reviews contained clinical data for segmented contacts used in ring mode. In this case, we estimated the corresponding electrophysiological features by averaging band-limited power and coherence across ring segments.

#### Therapeutic window

To account for variability in the review procedure, such as variable step-size and maximum amplitude, we normalized the therapeutic window such that it ranged between −1 and 1, with 1 describing the best-case scenario (clinical effects at lowest amplitude, first side effects at highest amplitude or not at all) and −1 describing the worst-case scenario (side effects at lowest amplitude, first clinical effects at highest amplitude or not at all). This was achieved by dividing the therapeutic window by the range of amplitudes tested. Missing values, indicating that no effect occurred up to the highest amplitude tested, were replaced by the maximum amplitude tested. The procedure is illustrated in Table 3. Using higher values than the maximum amplitude tested for replacement did not impair model performance (Supplementary Table S4).

**Table 3:** Therapeutic window normalization

Before normalization					After normalization			
Contact	SET [mA]	TET [mA]	TW [mA]		SET [mA]	TET [mA]	TW [mA]	TW (*z*) [mA]
**1**	3	1	2	→	3	1	2	0,34
**2**	—	1	—	→	**6**	1	**5**	**0,85**
**3**	4	—	—	→	4	**6**	**-2**	**-0,34**
**4**	2,5	1	1,5	→	2,5	1	1,5	0,25

The table illustrates monopolar review data before (left) and after normalization (right). First, we replaced missing values by the maximum amplitude tested (6 mA in this case). Next, we divided all windows by the range of amplitudes tested (5.9 mA in this case).

*SET* side-effect threshold, *TET* therapeutic effect threshold, *TW* therapeutic window, *TW(z)* normalized therapeutic window.

#### Model

**Fig. 5:**
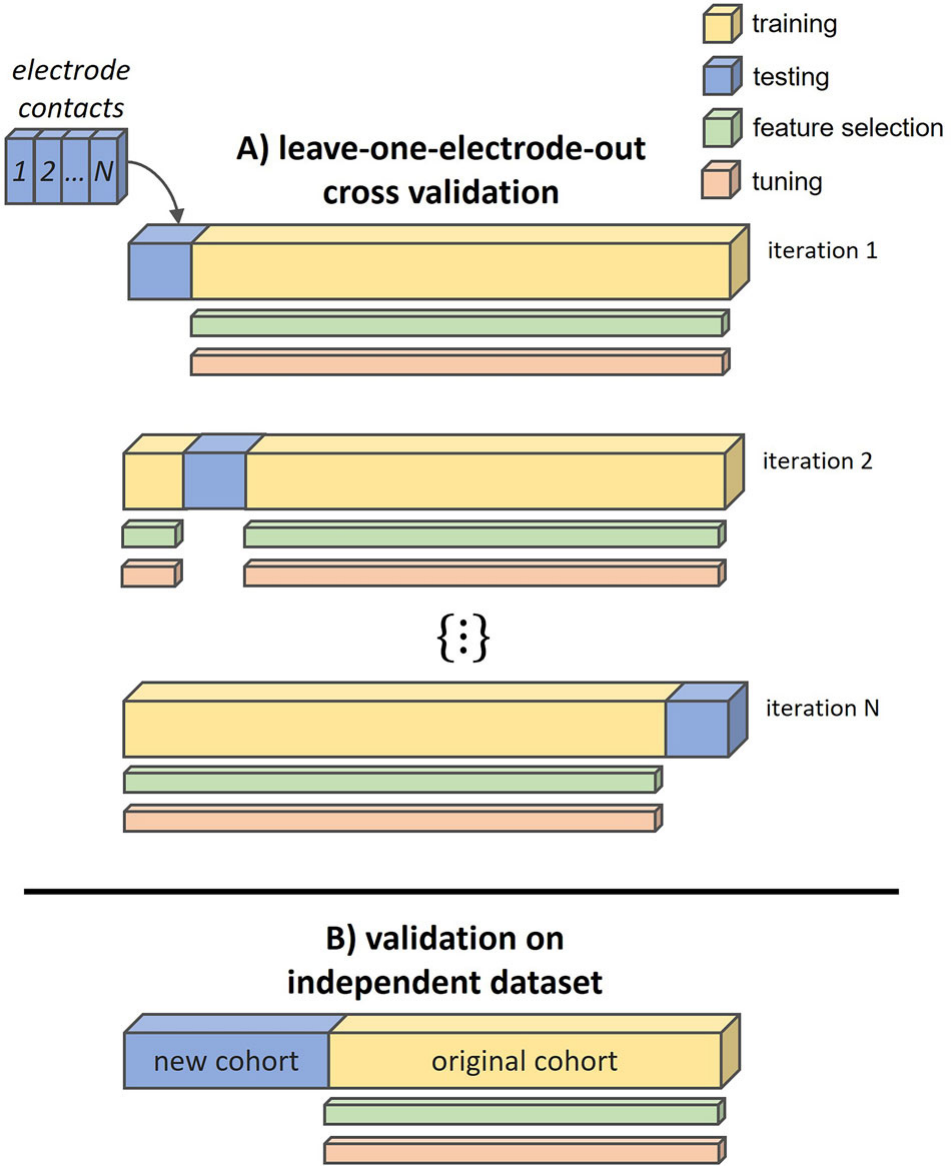
Model evaluation pipeline. **A** Leave-one-electrode-out cross-validation. For each iteration, one electrode from the original cohort (blue) is left out for testing, while the remaining electrodes (yellow) are used for training, including nested feature selection and hyperparameter tuning. This process is repeated for every electrode (*N* = 82) in the dataset. Once all electrodes are evaluated, the performance metrics are aggregated to assess the model’s overall performance. **B** Validation in an independent cohort. A model trained on the entire original cohort (yellow) is applied to a new cohort (blue).

We used extreme gradient boosting, as implemented in the XGBoost package for Python^44^, to predict the therapeutic window of each electrode contact based on STN power and STN-cortex coherence. This machine learning approach has been widely used and proven effective in analysing electrophysiological datasets^56,84^. As illustrated in Fig. 5A, predictions were iteratively generated for each electrode using a LOEO cross-validation approach, wherein each electrode served as the test set once while being included in the train set for all other iterations. In terms of hyperparameters, we first selected the number of trees by means of 10-fold cross-validation and then applied the Hyperopt package^85^. Tuning was nested, i.e., restricted to the train set. The selected hyperparameters, averaged across LOEO cycles, are reported in Supplementary Table S1.

#### Feature selection

During each iteration of the LOEO cycle, we utilized the Boruta method for nested feature selection^86^. Boruta works by first creating a copy of the original features, called “shadow features”, with values randomly shuffled to destroy any correlation with the target. A Random Forest model is then trained on the combined set of original and shadow features, producing an importance metric for each feature. Original features scoring significantly lower (*p* < 0.05 in a binomial test) than the most important shadow feature are removed. Boruta repeats this process for a maximum of 100 iterations or until all features are either confirmed or rejected, removing more unimportant features in each iteration. To ensure a robust set of features across iterations, we resampled from the train set and applied the Boruta method 10 times, keeping only features that were selected ≥7 times. The selection frequency, i.e., the fraction of LOEO cycles preserving a feature, served as our feature importance metric.

#### Model evaluation

The model’s performance was quantified by Pearson’s correlation between the predicted and actual therapeutic windows. Additionally, we assessed the model’s ability to rank electrode contacts by clinical utility—a potential use case for our method. Specifically, we ranked the contacts electrode by electrode based on their predicted therapeutic window, assigning higher ranks to contacts with higher predicted windows. We then computed the cumulative hit ratio, quantifying how often the active contact, i.e., the clinician’s choice, coincided with contact ranked first, was among the top two ranks, the top three ranks and so forth. As more and more contacts are considered, the cumulative hit ratio increases, eventually reaching 100% because the active contact must be among the set of all available contacts. Next, we computed an empirical null distribution by randomly ranking contacts 10,000 times and re-computing the cumulative hit ratio. Ratios exceeding the upper 95th percentile of the null distribution were deemed significant. Note that we limited this analysis to electrodes with data for at least four contacts (*N* = 47), to ensure a reasonable number of unique within-electrode permutations.

To further test the generalizability of our model, we re-trained it on the entire, original cohort (45 patients), including feature selection, and applied it to the independent cohort of eight PD patients, not available to the model during training. The selected hyperparameters of this model are summarized in Supplementary Table S1.

## Supplementary information

Supplementary Information

## Data Availability

The data from LOEO cohort 1 are available at OpenNeuro.org with the accession number ds004998 (10.18112/openneuro.ds004998.v1.2.2). The remaining data were not publicly available because patients did not consent to data sharing.

## Code availability

The code for this study is publicly available at https://github.com/Fayed-Rsl/DBS_Prediction_TW.
