# Supplementary material for: Electrophysiological signatures predict the therapeutic window of deep brain stimulation electrode contacts
Source: NPJ Digit Med. 2025 Oct 29;8:635. doi: 10.1038/s41746-025-02089-w (PMC12572235; doi:10.1038/s41746-025-02089-w)
Supplement: Supplementary file 1 — Supplementary Information [file 41746_2025_2089_MOESM1_ESM.pdf]

Electrophysiological signatures predict the therapeutic window of deep brain stimulation electrode contacts

Supplementary Material

Fayed Rassoulou[1], Abhinav Sharma[2,3], Alexandra Steina[1], Markus Butz[1], Christian J. Hartmann[1, 4], Bahne H. Bahners[1,4], Jan Vesper[5], Alfons Schnitzler[1, 4], Jan Hirschmann[1]

Author affiliations

1. Institute of Clinical Neuroscience and Medical Psychology, Medical Faculty and University Hospital Düsseldorf, Heinrich Heine University Düsseldorf, Germany
2. MRC Brain Networks Dynamics Unit, University of Oxford
3. Nuffield Department of Clinical Neurosciences, University of Oxford
4. Center for Movement Disorders and Neuromodulation, Department of Neurology, Medical Faculty and University Hospital Düsseldorf, Heinrich Heine University Düsseldorf, Germany
5. Department of Functional Neurosurgery and Stereotaxy, Medical Faculty and University Hospital Düsseldorf, Heinrich Heine University Düsseldorf, Germany

Hyperparameter tuning

Table S1 summarizes the hyperparameters used in the leave-one-electrode-out (LOEO) cycle and for predicting the therapeutic windows of an independent cohort. Parameters in orange were tuned using the Hyperopt package within a nested, 3-fold cross-validation framework. For the LOEO cycle, the table provides the hyperparameters averaged across iterations. For the independent cohort, the hyperparameters were found through a cross-validated search in the entire original cohort. Parameters not tuned (black) remained at their default settings.

Table S1. Optimized hyperparameters for XGBoost.

	**LOEO cycle**	**indep. cohort**
**parameter name**	**value [average]**	**value**
objective	reg:squarederror	reg:squarederror
base_score	0.5	0.5
booster	gbtree	gbtree
colsample_bylevel	1	1
colsample_bynode	1	1
**colsample_bytree**	**0.93**	**0.81**
**gamma**	**0.08**	**0.07**
learning_rate	0.05	0.05
max_delta_step	0	0
max_depth	7	7
**min_child_weight**	**4**	**4**
missing	None	None
**n_estimators**	**315**	**286**
**reg_alpha**	**0.18**	**0.19**
**reg_lambda**	**0.61**	**0.62**
scale_pos_weight	1	1
**subsample**	**0.62**	**0.51**

Parcellation

Table S2 lists the labels of the cortical parcels used in this study, along with the x, y and z coordinates of the grid point cloud centroids in MNI space.

Table S2. ***Details on cortical areas*.**

area	x	y	z
SensorimotorR	38.28	-14.42	54.59
FrontalSupR	17.78	33.48	41.75
FrontalMedR	36.35	38.63	20.92
FrontalInfR	52.30	31.11	1.94
ParietalSupR	23.00	-60.67	64.84
ParietalInfR	52.73	-47.50	47.29
TemporalSupR	63.47	-13.85	3.22
TemporalMidR	59.85	-23.91	-11.06
TemporalInfR	58.30	-35.63	-24.14
OccipitalSupR	19.94	-94.60	24.55
OccipitalMidR	39.38	-85.00	17.80
OccipitalInfR	29.33	-95.00	-9.09
AngularR	47.81	-65.00	39.75
SupraMarginalR	61.83	-32.50	34.73
CerebellumR	33.90	-61.22	-46.50
SensorimotorL	-39.35	-12.95	54.26
FrontalSupL	-14.80	44.73	30.35
FrontalMedL	-32.91	40.96	24.72
FrontalInfL	-50.60	29.52	3.11
ParietalSupL	-17.14	-61.76	64.62
ParietalInfL	-49.08	-51.11	46.36
TemporalSupL	-59.66	-8.18	-1.40
TemporalMidL	-61.87	-31.36	-5.78
TemporalInfL	-59.29	-28.33	-25.71
OccipitalSupL	-8.64	-97.80	21.46
OccipitalMidL	-33.41	-91.75	10.92
OccipitalInfL	-24.92	-91.83	-10.07
AngularL	-48.73	-66.00	36.52
SupraMarginalL	-61.99	-33.33	30.51
CerebellumL	-31.65	-61.54	-46.86

Reduced Feature Sets

One way of addressing feature importance is to exclude a subset of features and to re-assess prediction performance. Here, we used this strategy to investigate the contribution of STN power vs.

STN-cortex coherence, the importance of the cerebellum, and the possibility to obtain accurate predictions when using only subthalamic and primary sensorimotor features.

A model trained on STN power alone yielded a lower performance than the full model (r = 0.22, p < 0.001; **Fig. S1A**). In contrast, the STN-cortex coherence model achieved a correlation comparable to the full model (r = 0.40, p < 0.001; **Fig. S1C**). Despite the relatively good prediction, the cumulative hit ratio analysis did not indicate any ability to speed up the search for the optimal contact (**Fig S1D**), emphasizing the advantage of combining STN power with STN-cortex coherence.

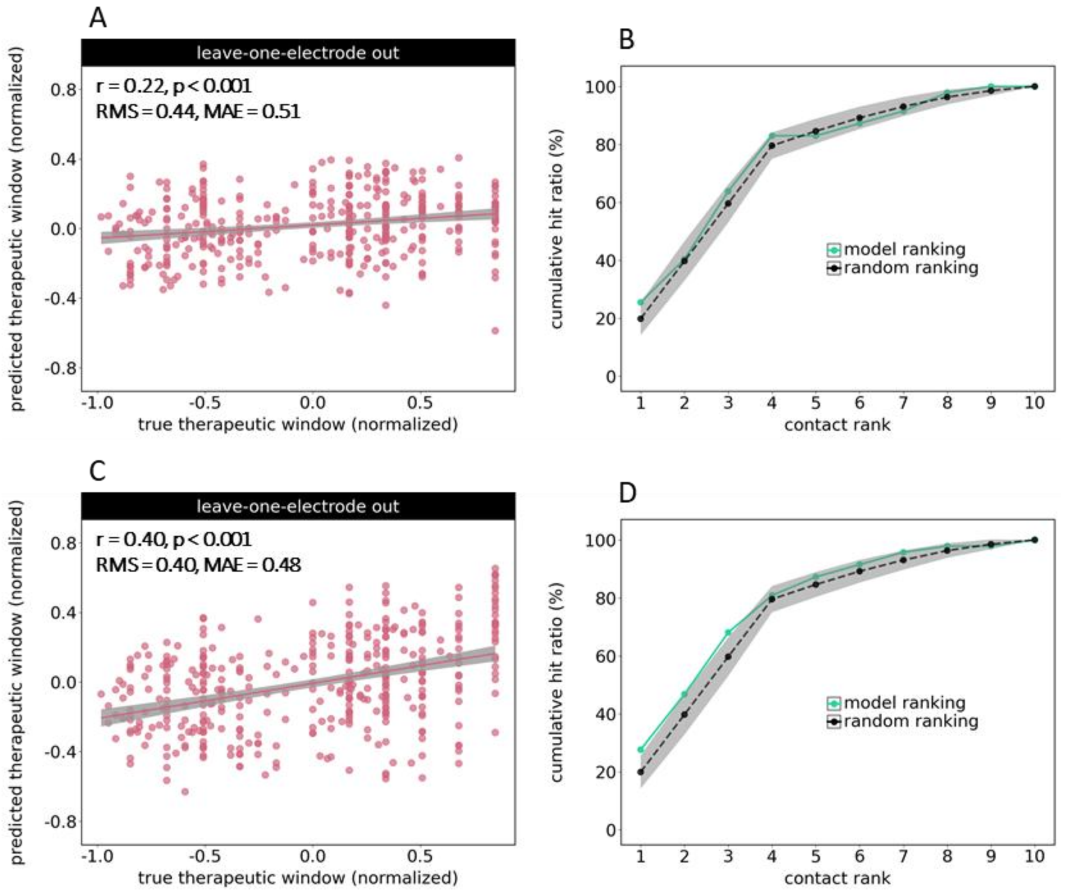

***Figure S1. STN-power-only and STN-cortex-coherence-only feature sets*.** **A)** STN-only set. Scatter plots displaying the relationship between actual and predicted therapeutic windows for the leave-one-electrode-out approach. Grey shaded areas represent 95% confidence intervals. **B)** STN-only set. Cumulative hit ratio for the model's ranking of DBS electrode contacts (green) and the average hit ratio resulting from random ranking (black). The hit ratio at any rank, say 3, reflects the fraction of electrodes for which the active contact got ranked 3rd or better. Grey shaded area represents mean ± 1 standard deviation. Asterisks (*) indicate above-chance performance (p < 0.05). **C)** Scatter plot for coherence-only feature set. **D)** Cumulative hit ratio for coherence-only feature set.

We tested two further reduced feature sets that lacked some of the brain areas included in the original analysis. The first area-reduced feature set included all brain areas except the cerebellum (**Fig. S2A**). Feature selection was switched on, as in the original analysis. The second feature set, termed "ECoG" set, comprised only STN power and STN-sensorimotor coherence (all frequency bands). We used it to estimate the predictive power of a spatially restricted setup, mimicking the combination of STN electrodes and motor cortical electrocorticography (**Fig. S2B**). Automated feature selection was switched off. In both cases, the predicted therapeutic windows correlated significantly with the actual therapeutic windows, but the hit ratio analysis did not indicate a capacity to accelerate the search for the optimal contact (**Fig. S2**).

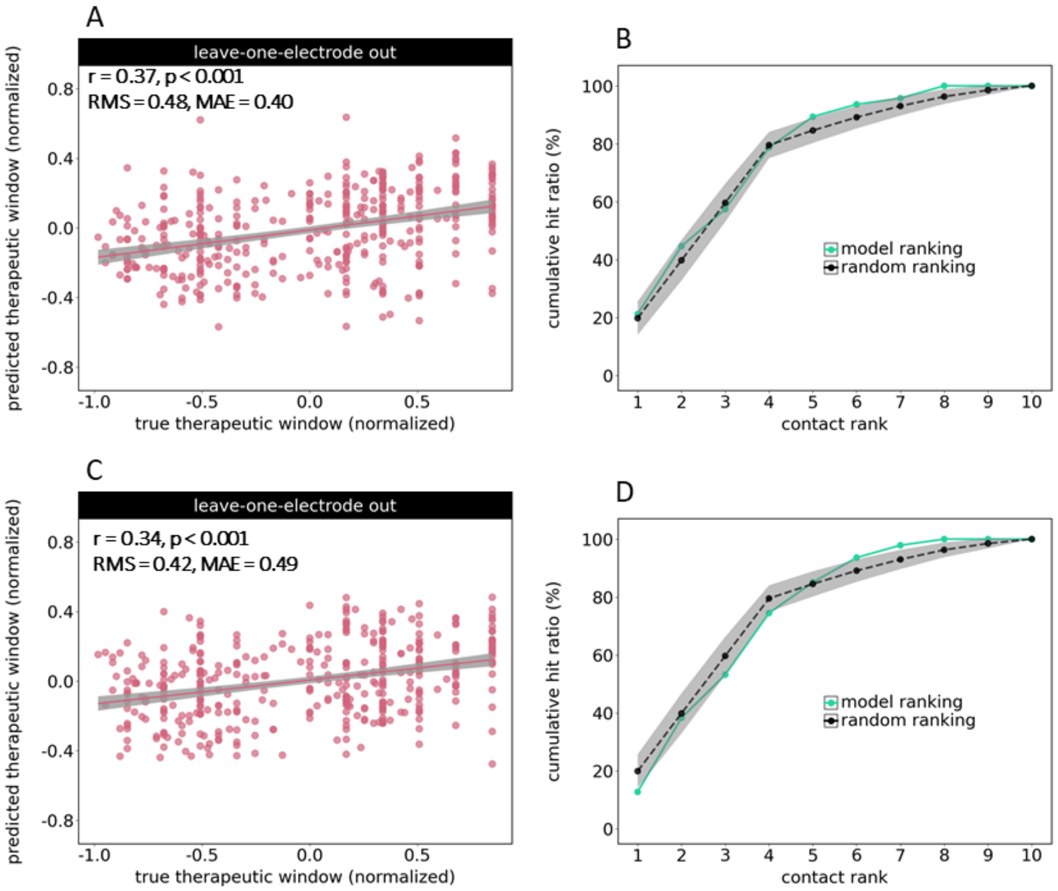

Figure S2. Area-reduced feature sets. **A,B)** All features except cerebellum. **C,D)** "ECoG" feature set (STN and sensorimotor cortex). See Fig. S1 for more explanations.

Interestingly, the algorithm relied much more on STN power features when excluding the cerebellum (**Fig. S3**).

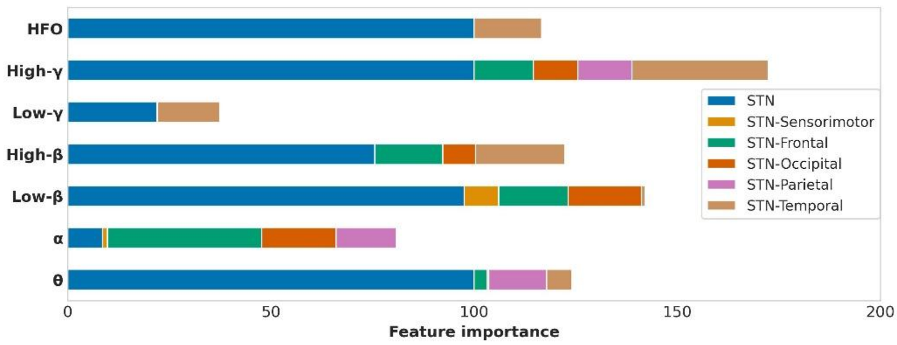

Figure S3. Feature importance for the feature set lacking the cerebellum. The bar plot illustrates the importance of each feature, grouped by brain region (indicated by colors) and frequency band (θ, α, β, γ, HFO). The height of each bar represents the relative contribution of each feature. Feature importance was quantified by selection frequency across leave-one-out cycles, normalized by the number of brain areas per lobe. Compare to Fig. 2 of the main paper.

Enhanced Feature Set

In order to test whether the addition of anatomical information would improve predictions we tested an enhanced feature set that included the best-performing electrophysiological features (main paper, **Fig. 2A**) and the distance of the contacts' centre to a published anatomical sweet spot for STN DBS (x = 12.58, y = -13.41, z = -5.87; Horn et al., Hum Brain Mapp **38**, 3377–3390, 2017). This required electrode reconstruction, conducted with LeadDBS (Horn & Kühn, Neuroimage 107:127–135, 2015), based on presurgical T1 and T2 MRI scans and postsurgical CT.

Fig. S4 illustrates the correlation between predicted and actual therapeutic windows without (**Fig. S4A**) and with (**Fig. S4C**) the additional anatomical feature, as well as the cumulative hit ratios (**Fig. S4B and S4D**).

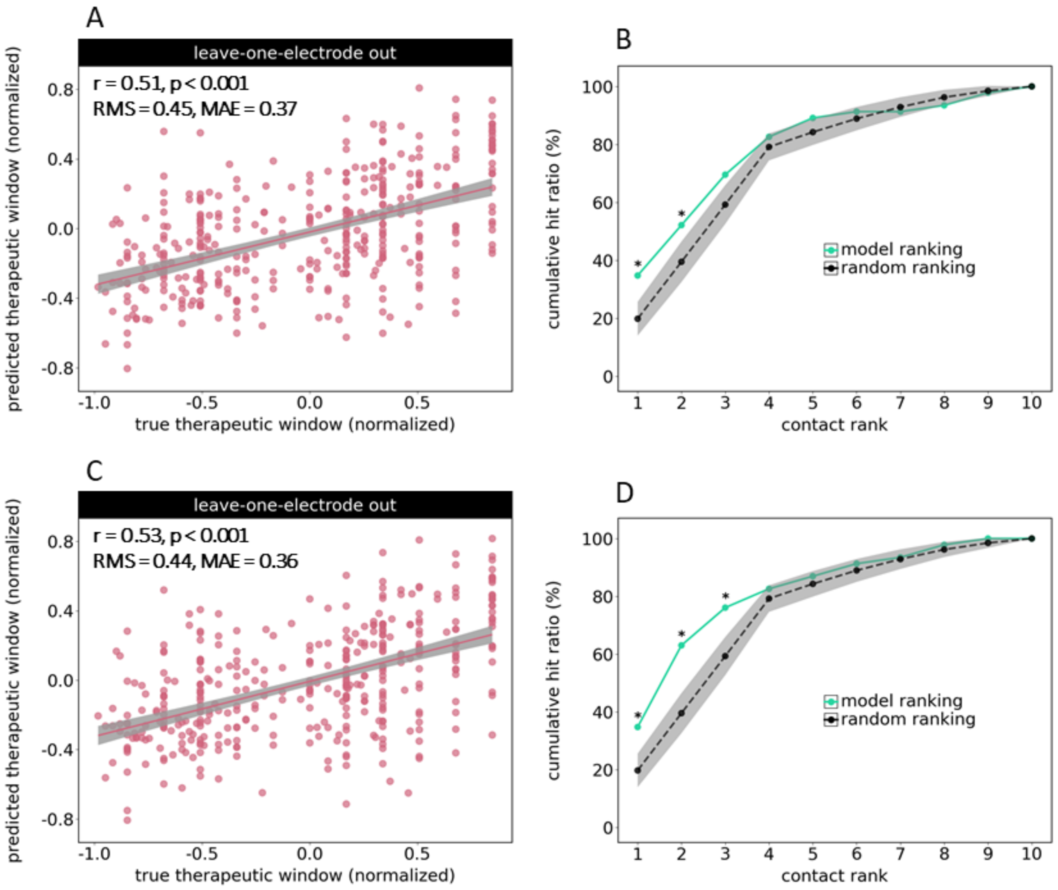

Figure S4. Enhanced feature set. **A,B)** Best-performing electrophysiological features. **C,D)** Best-performing electrophysiological features plus distance to sweet-spot. See Fig. S1 for more explanations.

Linear Mixed Model

Pearson correlation, as used in the main performance analysis, treats the contacts as independent samples and neglects their hierarchical structure. To confirm that this limitation does not confound our interpretation, we used a Linear Mixed Model as an alternative performance measure, with the following formula:

$$true \sim pred + (1|hemisphere)$$

where:

- **true** represents the actual therapeutic window.
- **pred** corresponds to the predicted therapeutic window.
- **(1|hemisphere)** is a random intercept for each hemisphere. Hemispheres are assumed to be independent samples randomly drawn from a large population.

The results presented in **Table S3** demonstrate a significant relationship between predicted and actual values (β = 0.312, p = 0.005), while accounting for repeated measures across hemispheres/electrodes.

Table S3. Fit results for the Linear Mixed Model estimating the relationship between predicted and actual therapeutic window. The positive coefficient for *pred* indicates a significant relationship between predictions and actual values.

fixed effects	coefficient (β)	std. error	*z*-value	*p*-value	95% CI (lower)	95% CI (upper)
intercept	-0.012	0.048	-0.250	0.802	-0.106	0.082
pred	0.312	0.112	2793	0.005	0.093	0.532

random effects	variance	std. dev.
hemisphere (intercept)	0.171	0.130

Reduced samples

In our train dataset, only 28 of 45 patients exhibited clear subthalamic power peaks in the beta range. To investigate whether and how the presence of such a peak influences automated feature selection, we repeated the feature selection process in the subset of patients exhibiting clear beta peaks. As depicted in Fig. S5, this led to the selection of STN alpha and low-beta power instead of gamma and HFO power, which were the most important STN features in the entire dataset.

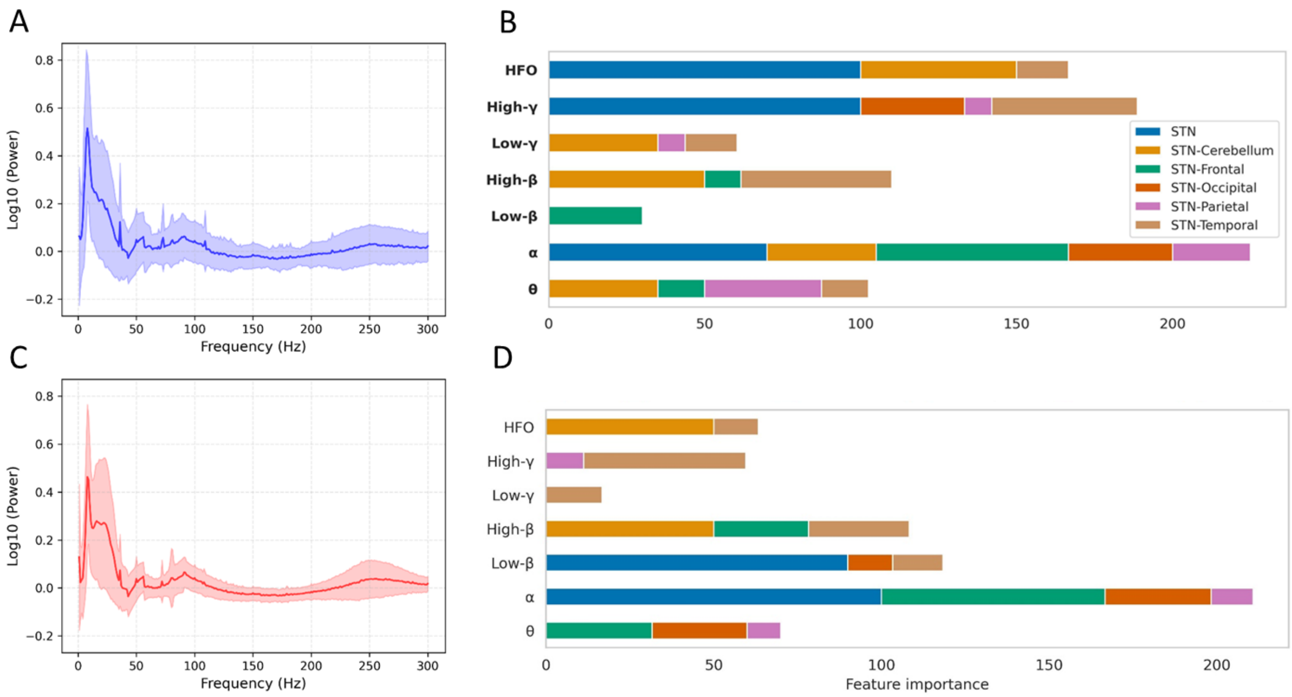

***Figure S5. Feature importance depends on peak availability*. A)** Group-mean subthalamic power spectrum and standard deviation (shading). All patients included. **B)** Feature importance. All patients included. **C)** As A), but includes only patients with a clear subthalamic power peak. **D)** As B), but includes only patients with a clear subthalamic power peak.

Data Imputation

In this study, we replaced missing clinical or side-effect thresholds with the highest amplitude tested to indicate that no side-effect or no clinical effect was observed up to that point. We acknowledge that the true thresholds are not known in these cases, but we do know that they must actually be larger than the highest amplitude tested. In order to investigate whether assuming higher thresholds would affect our conclusions, we systematically increased the imputed values by 2mA in steps of 0.5mA and re-evaluated model performance in leave-one-electrode-out cross-validation. The correlation between predicted and actual therapeutic windows was hardly affected by this change, indicating that the exact value used for replacing missing values is not critical to model performance.

Table S4. Increasing the value used in data imputation did affect model performance. The correlation coefficient quantifies the correlation between actual and predicted therapeutic windows in leave-one-electrode-out cross-validation.

**value used for replacement**	**Pearson correlation**
max. amplitude tested	0.44
max. amplitude tested + 0.5mA	0.43
max. amplitude tested + 1.0mA	0.44
max. amplitude tested + 1.5mA	0.44
max. amplitude tested + 2.0mA	0.43
